# Occurrence and temporal changes of pharmaceuticals in the Warta River in Poland during and after the COVID-19 pandemic

**DOI:** 10.1038/s41598-025-14568-x

**Published:** 2025-08-12

**Authors:** Roksana Kruć-Fijałkowska, Dariusz Drożdżyński, Magdalena Matusiak, Krzysztof Dragon, Marek Szczepański

**Affiliations:** 1 Hydrogeology and Water Protection Unit, Institute of Geology, Adam Mickiewicz University in Poznań, Bogumiła Krygowskiego 12, Poznań, 61‑680 Poland; 2 Department of Pesticide Residue Research, Institute of Plant Protection-National Research Institute, Władysława Węgorka 20, Poznań, 60‑318 Poland

**Keywords:** River water contaminants, Pharmaceuticals, Sewage treatment plants, COVID-19 waves, COVID-19 reopenings, Environmental sciences, Hydrology

## Abstract

This research aimed to assess the impact of the COVID-19 pandemic on pharmaceutical concentrations in surface water. The Warta River (western Poland) was studied at points upstream and downstream of the treated sewage outflow. Ten pharmaceuticals were analyzed in 12 sampling campaigns during the pandemic (February 2020 - April 2021), and 3 during the post-pandemic period (November 2024 - January 2025) to assess their long-term impact. The results revealed a significant increase in pharmaceutical concentrations near the outflow of treated sewage (range 1.53–20.47 µg/L), confirming that it is the main source of these micropollutants in the environment. Moreover, the concentration increase in the river, overlapped with successive waves of the COVID-19 pandemic. This made it possible to identify patterns of society’s behavior. During lockdowns, preventive and psychotropic drug concentrations increased in the river. These indicate that higher consumption of these pharmaceuticals maybe associated with worse mental well-being and a desire to protect against the virus. However, the concentrations of antibiotics and cardiological drugs decreased, which was linked to more difficult access to doctors and protection from the virus by avoiding the health center. During the post-pandemic period psychotropic drug concentrations increased significantly, suggesting a significant deterioration in society’s mental health.

**Supplementary Information:**

The online version contains supplementary material available at 10.1038/s41598-025-14568-x.

## Introduction

Water pollution is an escalating global problem, with rivers being the most affected natural water resources. The quality of surface water is threatened by various pollutants, including emerging contaminants^1,2^. Worldwide, data have revealed the occurrence of heavy metals (e.g. cadmium, lead, mercury)^3^, pesticides (herbicides, insecticides and fungicides)^4–6^, pharmaceuticals (e.g. antibiotics, hormones, psychoactive substances)^7–9^ and even microplastics^10^.

One of the most serious threats is pharmaceuticals, owing to their widespread presence and persistence in aquatic environments. These micropollutants have been reported worldwide in surface water, especially in rivers where treated sewage is discharged^11,12^. The sewage treatment process cannot fully remove all contaminants; thus, pollutants are released into the aquatic environment^13–15^. Municipal effluents are considered the most significant suppliers of pharmaceuticals to the aquatic environments^16–18^. Commonly detected compounds in this group of micropollutants include antibacterials, endocrine disruptors, antiepileptic agents, antidepressants, analgesics and antifungals^8,19,20^.

In 2019, humanity faced a great challenge: the global COVID-19 pandemic. The almost 3-year-long pandemic has significantly impacted people and changed their current lives, customs and habits^21–23^. People were forced to stay at home, relying on online work and education, with minimal opportunities for in-person entertainment and social interactions. Difficulties in adapting to new circumstances, accompanied by an emerging sense of insecurity, can lead to serious negative consequences for psychological functioning^24,25^. One of the most difficult experiences of the ongoing pandemic was the death of many people. According to several reports from different countries, a significant percentage (17–46%) of the population has experienced psychological distress following the COVID-19 outbreak^26–28^. It is undeniable that the COVID-19 pandemic has significantly impacted people’s lives and activities.

The main cause of presence of the pollution in the compartments of the environment is human activity. The COVID-19 pandemic has had a considerable impact on people’s lives and behaviors by, changing their previous activities. It is therefore probable that some changes occurring during this period were also visible in the environment, as confirmed by the literature^29,30^. The environmental impact of the pandemic has been noticeable in terms of climate, air, and water quality^31–33^. The impact is also evident in the quality of surface water^34–36^. In a study conducted in Poland, Luczkiewicz et al.^37^ reported changes in the physical and chemical parameters of raw wastewater caused by behavioral and social changes, including altered water consumption patterns, reduced activity in the tourism sector and increased use of disinfectants. However, the changes that occur in the environment during the pandemic are not always negative^38^. Uddit et al.^33^ reported that water quality indicators, which have remained within permissible limits over the years, significantly decreased during lockdowns. The dissolved inorganic nitrogen and total organic nitrogen decreased, and the water transparency improved. Studies performed in the Damodar River, India, also revealed improvements in water quality (a decrease in heavy metal concentration) during lockdowns^39^. Other studies have indicated improvements in water quality during lockdowns because of less human activity^40–42^. Tourist traffic limitations during the pandemic resulted in a significant improvement in beach conditions^34,35^.

The fight against a serious disease, such as COVID-19 requires the use of large amounts of pharmaceuticals. The outbreak of the COVID-19 pandemic could affect the composition and loads of these micropollutants in treated sewage. The consumption of pharmaceuticals used to fight the pandemic, as well as preventive drugs (anti-inflammatory drugs, painkillers), has increased^43,44^. This is supported by statistical data on the sales of painkillers in Poland^45^. Their metabolites are released into the environment, including water and soil^46,47^. Therefore, connections between the pandemic and the occurrence of pharmaceuticals in the environment should be investigated.

Data from the literature suggest that pharmaceutical concentrations in surface water are significantly influenced by the COVID-19 pandemic^43,47^. Additionally, monitoring of pharmaceuticals in water is considered crucial during pandemics^48^. Most of the published data regarding the pandemic period and the presence of pharmaceuticals in river water are based on single-period or short-term studies^34,49–52^. Therefore, it is not possible, or only minimally possible, to analyze the impact of tightening and easing restrictions on human behaviour and the environment, as well as changes occurring between lockdowns. One study analyzed 16 research campaigns, covering two lockdowns^53^. Nonetheless, in the literature, information on the spectrum and concentration of substances present in surface water is limited, and this should be the subject of further research^43^.

In May 2023, the World Health Organization declared the end of the public health emergency, leading to the discontinuation of lockdowns and restrictions. The population subsequently resumed prepandemic activities. Although a complete return to prepandemic behavioral patterns was unlikely - given the profound impact of the lockdowns on many individuals-human activity was undoubtedly underwent a process of change once again. One particular intriguing aspect is how the shifts in human activity manifested in the aquatic environment. To determine whether the COVID-19 pandemic has caused any lasting changes, studies in the same location during both the pandemic and post-pandemic periods should be performed and compared. The literature data comparing these two timeframes are limited^54^.

The objectives of the research were: (1) to assess the impact of the COVID-19 pandemic on the presence of pharmaceuticals in river water; (2) to investigate the relationship between the concentration of micropollutants and the implementation and easing of pandemic restrictions (including three subsequent lockdowns); (3) to compare the pharmaceutical concentrations in river water during the pandemic period with those during the post-pandemic period; and (4) attempts to determine societal behavior, mental health, and physical well-being, based on river water analysis.

## Materials and methods

### Site description and sampling

**Fig. 1:**
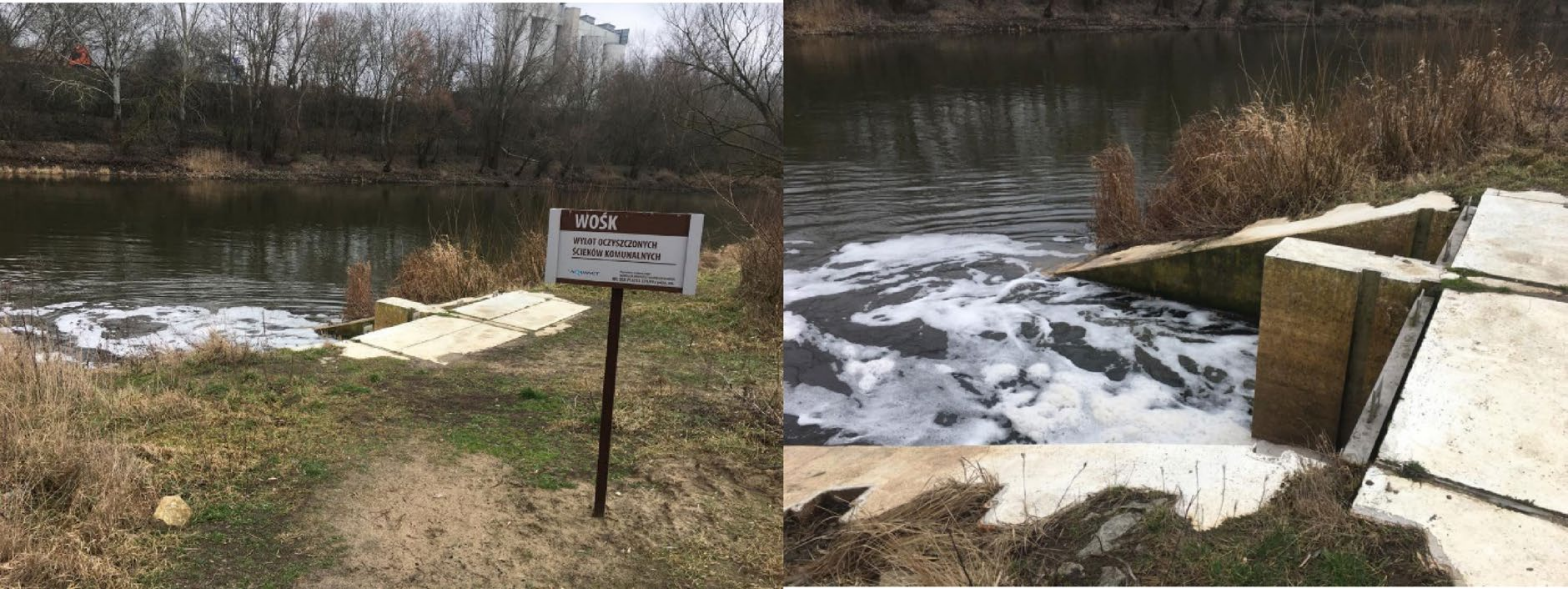
Treated sewage discharges into the Warta River.

**Fig. 2:**
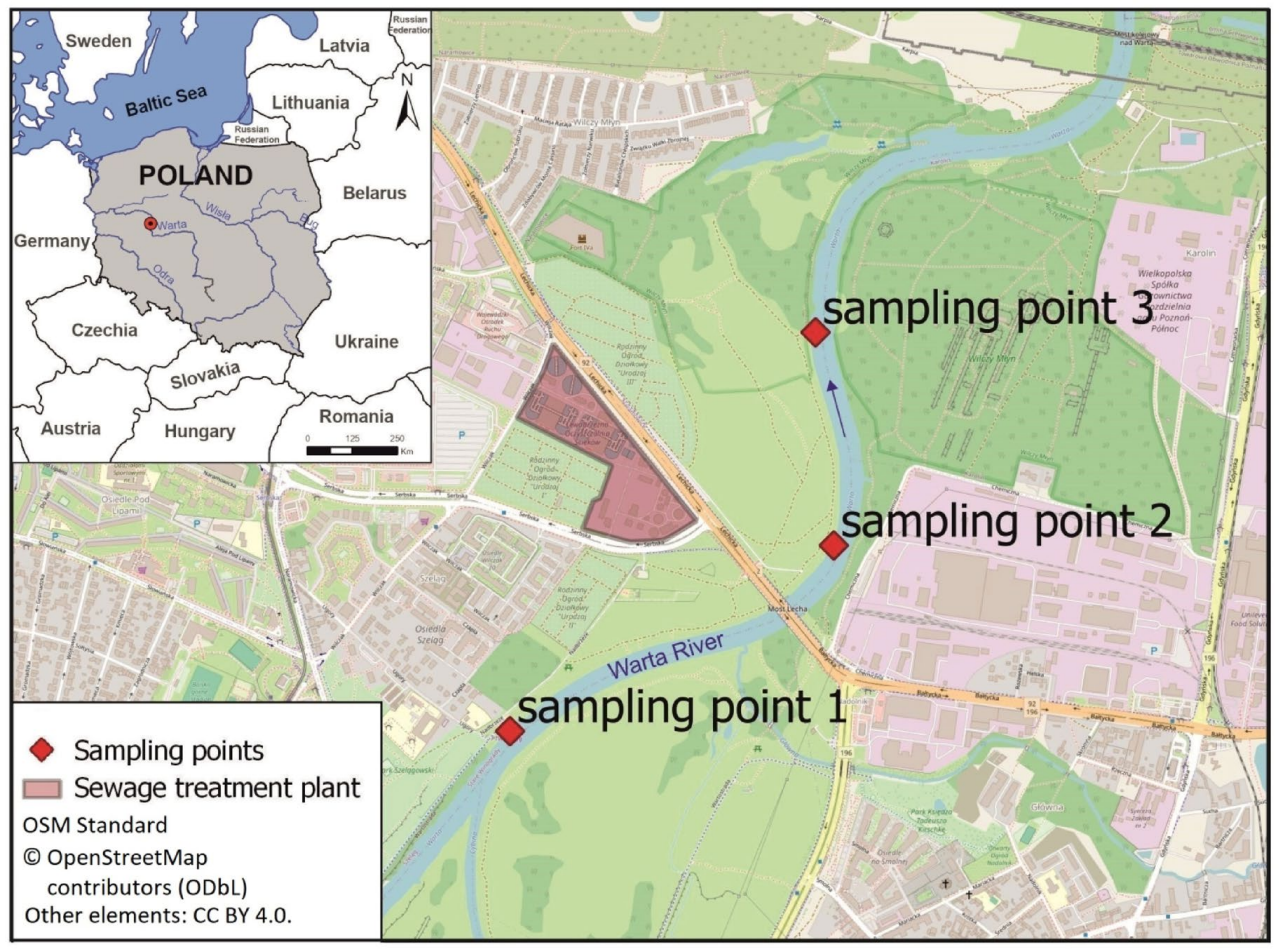
Locations of the treatment plant and sampling points. The map was created using QGIS 3.40.7 (QGIS Development Team, https://qgis.org) with the OpenStreetMap Standard basemap (©OpenStreetMap conributors, licensed under the Open Database License (ODbL): https:/www.openstreetmap.org/copyright). All original elements of the figure are available under the CC BY 4.0 license.

The research was performed in the area of Poznań city, western Poland. The neighbourhood of the Left-bank Sewage Treatment Plant, which collects municipal sewage, was selected for the study (Fig. 1). It is a mechanical (primary treatment) and biological (secondary treatment) sewage treatment plant designed for increased removal of nutrients and full processing of the generated sewage sludge. During the primary step of sewage treatment, aerated sand separators are applied to separate the sand and other solids from the water. The secondary treatment is biochemical oxidation, which uses aerobic microorganisms and applies simultaneous ammonification, nitrification, and denitrification processes to remove nitrogen. In parallel dephosphatation is used for phosphorus removal. This process is supported by the use of the gel coagulant PIX. After this process, the treated sewage flows through a measuring chamber to the Warta River^55^. The treatment plant serves 270,000 inhabitants, and the facilities are adapted to receive maximum 50,000 m^3^ of sewage per day. The treated sewage is discharged to the Warta River approximately 350 m away (in a straight line) directly southeast of the sewage treatment plant (Fig. 2).

The Warta River is the third longest river in Poland. The length of the river is 808 km, and the catchment area is 54,519 km^2^^56^. The area features a varied landscape, stretching from highlands in the south, through the central lowlands, to the lake district zone in the north^57^. Data from water gauges located on the Warta River in Poznań city were used in the study. River levels during the research period from 2020 to 2021 fluctuated from 114 cm to 311 cm, whereas flowrates ranged from 24 m^3^/s to 139 m^3^/s.

Water samples directly from the Warta River were obtained for the tests. Three sampling points were selected. One of them (sampling point 1) was river water from a place not exposed to the impact of a sewage treatment plant (sampling point – upstream of the treated sewage outflow). Two of them were exposed to the impact of the wastewater treatment plant: sampling point 2 (river water at the location of the outflow of treated sewage from the sewage treatment plant) and sampling point 3 (river water from a distance of approximately 500 m from the location where the treated sewage is discharged into the river) (Fig. 2). A total of fifteen sampling campaigns were performed in two periods: twelve sampling campaigns from February 2020 to April 2021, and three sampling campaigns from November 2024 to January 2025. During each sampling campaign, one water sample was collected from each of the indicated points.

River water samples were taken from the river bank using a 3 m telescopic bucket (Telescoop) at a depth of 30 cm. The samples were collected into 1 L brown glass bottles, which were protected from UV light. The bottles were rinsed three times with collected surface water. Water samples were not fixed and were transported to the laboratory under refrigerated conditions (4 °C).

### Chemical analysis

Raw samples of collected surface water were filtered through standard grade qualitative filter papers (Ahlstrom, Helsinki, Finland). The filtered sample (8 mL) was added to a 50 mL centrifuge tube. Then, 40 µL of internal standard (carbamazepine-D10, Dr Ehrenstorfer, Steinheim, Germany) was added at a concentration of 0.25 ng/mL, and the mixture was vigorously shaken by hand. After that, 10 mL of acetonitrile (LC gradient grade, Sigma-Aldrich, St. Luis, MO, USA) was added, and the mixture was shaken for 10 min (490 rpm, linear motion shaker, ChemLand, Stargard, Poland). The buffer mixture (Citrate Extraction Tube, Sigma-Aldrich, St. Luis, MO, USA) was subsequently added, and the mixture was shaken for 5 min on an orbital shaker (Multi Reax, Heidolph, Schwabach, Germany). The sample was subsequently centrifuged (4500 rpm, T = 15 °C, Rotina 420R, Hettich, Kirchlengern, Germany) for 3.5 min, after which 2.5 mL of acetonitrile extract was collected and transferred to a 15 mL centrifuge tube. The whole volume was evaporated to dryness under a gentle stream of nitrogen, and the dry residue was dissolved in 0.25 mL of acetonitrile and 0.25 mL of the LC phase (0.5% formic acid and 0.1% 1 M ammonium formate in water and 0.5% formic acid and 0.1% 1 M ammonium formate in methanol, 95:5, v; v, all solvents LC-MS grade, Merck, Darmstadt, Germany). The extract was transferred through a 0.22 μm syringe filter membrane (Millex^®^, Merck, Darmstadt, Germany) into chromatography bottles.

For the study ten different pharmaceutically active compounds (PhACs), i.e. carbamazepine (a psychotropic, anticonvulsant and mood stabilizing drug), diclofenac (an anti-inflammatory, analgesic and antipyretic drug), fluconazole (an antifungal), gabapentin (an antiepileptic and antianxiety drug), lamotrigine (an antiepileptic and a mood stabilizing drug), paracetamol (an analgesic), sulfamethoxazole (a human and veterinary antibiotic), sulfapiridine (an antibacterial), telmisartan (an antihypertensive) and tramadol (an analgesic, opioid) were selected based on previous research^7^. PhACs-certified reference materials were obtained from Dr. Ehrenstorfer Laboratory (Steinheim, Germany). All chemical compounds exhibited > 99% purity.

An LC-MS/MS system consisting of an Acquity Ultra Performance Liquid Chromatograph (Waters Corp., Milford, MA, USA) coupled to a QTRAP 6500 triple quadrupole mass spectrometer (AB Sciex Instruments, Foster City, CA, USA) was used for the analysis of the PhAC residues. A 10 µL aliquot was injected on Atlantis^®^ C_18_ (100 mm ×2.1 mm, 3 μm) column (Waters Corp.) at 40 °C. The gradient was composed of solvents A (0.5% formic acid and 0.1% 1 M ammonium formate in water) and B (0.5% formic acid and 0.1% 1 M ammonium formate in methanol) at a flow rate of 0.4 mL/min. The gradient elution was started with 5% B and increased linearly to 100% B in 4 min, then held at 100% for 1 min, and subsequently decreased to 5% A in 1 min and maintained at 5% B until the end of the run (6 min). The total time of data acquisition was 4.5 min. The MS/MS 6500 QTRAP was operated in electrospray ionization positive (ESI+) mode at a capillary voltage of 4500 V, a desolvation temperature of 400 °C, and an entrance potential (EP) of 10 V, with nitrogen as the curtain (CUR), nebulizer (GS1) and auxiliary (GS2) gas, at pressures of 50, 60 and 70 psi, respectively. Nitrogen was also used as a collision gas. The ionization and MS/MS collision energy settings were optimized while the pharmaceuticals solution was continuously infused at a 100 µL/min flow rate via a syringe pump. For each pharmaceutical, the parent ion and two or three daughter ions were selected. The first ion was used for quantification, and the others were used for confirmation. Data acquisition was performed in multiple reaction monitoring (MRM) mode. Analyst Software version 1.6.2 and MultiQuant Software version 3.0.2 (AB Sciex) were used for data acquisition and processing. The quantification level was determined to be 0.01 µg/L for all PhACs (Supplementary Tables S1-3).

### Statistical analysis

Statistical analysis was performed with Microsoft Excel and R studio^58^. One-way ANOVA tests were performed to determine significant differences (*p* < 0.05) between covariates, including sampling points and sampling time (COVID-19 wave or nonwave period). The correlations between the concentrations of pharmaceuticals and river water level, as well as precipitation, were determined. The Shapiro-Wilk tests was used to test the normal distribution of collected data (*p* > 0.05). Spearman’s correlation was selected based on the test findings.

### Stages of the COVID-19 pandemic in Poland

The coronavirus outbreak began in late 2019 in China and was declared a pandemic by the WHO in March 2020^59^. In the first period of the study (March 2020 – April 2021) more than 7% of the Polish population was infected^60^. During the pandemic, Poland experienced three waves of coronavirus, which the government combatted by alternately implementing and easing restrictions^49^.

The most restrictive bans, including pedestrian traffic restrictions and strict limits on the number of people attending gatherings, were in place during the first wave of the pandemic (the first lockdown) from April to the end of June 2020^61^, despite the low number of coronavirus cases detected (10–11 thousand per month). During the second lockdown (September-December 2020), which was introduced in response to a dramatic increase in the number of infections (24–606 thousand per month), the restrictions were less restrictive but still rigorous^62^. In contrast, during the third wave of coronavirus (March-April 2021), restrictions were weakest despite a significant increase in coronavirus cases (470–614 thousand per month). During the lockdowns collective childcare facilities were closed and home schooling was introduced. Most public institutions and a significant proportion of private companies adopted remote working policies. Shopping centres, tourist, sports and cultural facilities were closed and the principles of social distancing were applied.

Between the lockdowns (July-August and January-February 2021), when the number of coronavirus cases declined (to 11–22 and 194–218 thousand per month respectively), access to shopping centres, cultural and recreational facilities and entertainment venues was reinstated under strict sanitary conditions^63^.

The greatest easing of prohibitions was observed from January 2021 after the first COVID-19 vaccines were released^64^. Then, from March 2021, when the mass vaccination of the population begun, there was a noticeable increase in the number of workers returning to their offices. Finally, in May 2021, as the number of infections declined, most restrictions were lifted.

In the postpandemic period (November 2024 - January 2025), the number of new coronavirus cases was very low (4,500 per month), with a very low mortality rate^65^.

## Results

**Fig. 3:**
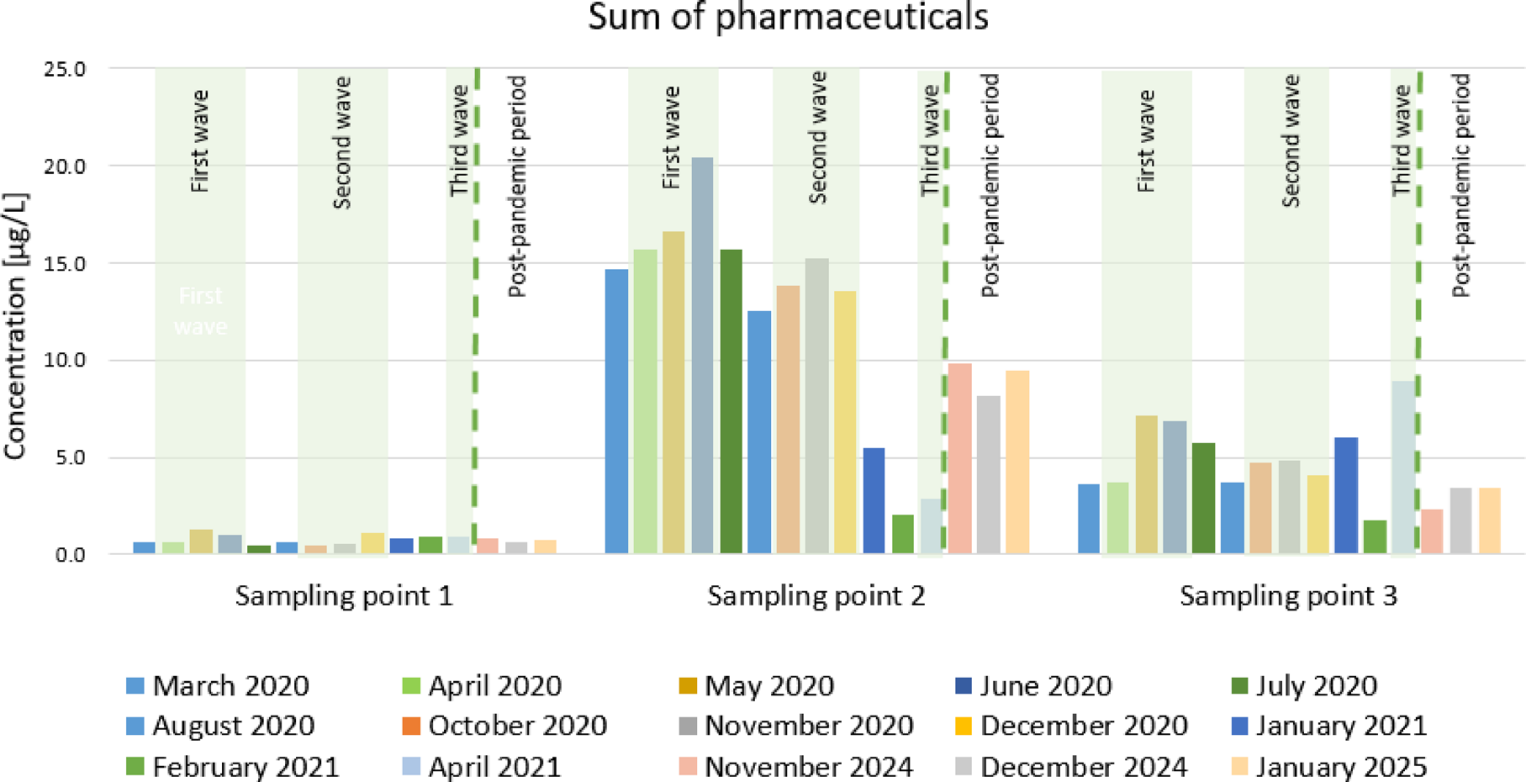
The sum of pharmaceutical concentrations in each sampling point in time.

The river water collected at Sampling point 1 (upstream of the treated sewage outflow) was characterized by low concentrations of the total pharmaceutical residues (0.48–1.26 µg/L). The total of the pharmaceutical concentrations noticeably increased at Sampling point 2 (2.02–20.47 µg/L). Then, at apoint approximately 500 m away, it again decreased to a value of 1.74–7.19 µg/L (Sampling point 3) (Fig. 3). ANOVA revealed the statistically significant differences in the concentrations of pharmaceuticals at the three sampling points (*p* < 0.05).

Among the individual pharmaceuticals, the highest concentrations at Sampling point 2 were diclofenac (7.51 µg/L, June 2020; 6.84 µg/L, December 2020), tramadol (6.43 µg/L, June 2020) and telmisartan (3.39 µg/L, April 2020) (Table 1). Gabapentin was detected seven times at levels above the LOQ, reaching a maximum of 0.09 µg/L (during the pandemic period) and a maximum of 0.80 µg/L during the post-pandemic period. Paracetamol was detected only once above the limit of quantification of 0.02 µg/L. Each of the pharmaceuticals showed variability over time.

**Table 1:** Pharmaceutical concentrations [µg/L] in the warta river at sampling point 2 (limit of quantification 0.01 µg/L), and data on precipitation and river water levels obtained from Institute of meteorology and water Management – National research Institute^66^ nd – no data.

Compounds	2020									2021			2024		2025
	III	IV	V	VI	VII	VIII	X	XI	XII	I	II	IV	XI	XII	I
	Onset	1st wave			Interwave		2nd wave			Interwave		3rd wave	Post-pandemic period		
Carbamazepine	0.73	0.82	0.80	0.96	0.61	0.47	0.58	0.59	0.61	0.54	0.19	0.35	0.73	0.64	0.73
Diclofenac	4.92	5.60	5.13	7.51	5.28	3.98	5.20	5.97	6.84	0.07	0.02	0.05	1.71	1.57	1.66
Lamotrigine	0.78	0.66	0.81	0.87	0.47	0.50	0.51	0.52	0.44	0.46	0.13	0.23	2.71	2.02	2.85
Fluconazole	1.06	0.96	0.79	0.88	0.77	0.70	0.83	0.55	0.48	0.34	0.09	0.15	0.18	0.16	0.32
Gabapentin	< LOQ	0.05	< LOQ	< LOQ	< LOQ	< LOQ	< LOQ	< LOQ	< LOQ	0.09	0.04	0.04	0.8	0.71	0.43
Paracetamol	0.02	< LOQ	< LOQ	< LOQ	< LOQ	< LOQ	< LOQ	< LOQ	< LOQ	< LOQ	< LOQ	< LOQ	< LOQ	< LOQ	< LOQ
Sulfametoxazole	0.09	0.19	0.09	0.06	0.55	0.56	0.57	0.40	0.13	0.19	0.07	0.11	0.31	0.27	0.22
Sulfapiridine	0.19	0.41	0.39	0.59	0.51	0.38	0.96	0.88	0.46	0.30	0.09	0.15	0.15	0.15	0.12
Telmisartan	2.65	3.39	3.38	3.17	2.62	2.35	1.59	1.30	1.34	< LOQ	< LOQ	0.01	1.62	1.26	1.56
Tramadol	4.20	3.61	5.23	6.43	4.93	3.64	3.60	5.03	3.07	3.08	0.90	1.45	1.62	1.38	1.56
Total pharmaceuticals	14.65	15.69	16.62	20.47	15.74	12.59	13.82	15.23	13.38	5.06	1.53	2.54	9.83	8.16	9.45
Monthly average precipitation in Poznań [mm]	29.4	1.7	46.6	52.9	67.9	59.6	39.9	11.9	21.7	55.3	35.9	33.5	31.9	18	44.9
Monthly average river water level in Poznań [cm]	188	149	139	135	133	118	203	252	209	250	280	227	nd	nd	nd

The highest concentrations of the total pharmaceuticals at Sampling point 2 were recorded in June (20.47 µg/L), May (16.62 µg/L), July (15.7 µg/L), April (15.69 µg/L) and November 2020 (15.23 µg/L). The lowest concentrations were observed in January (5.06 µg/L), April (2.54 µg/L) February (1.53 µg/L) 2021 (Table 1).

**Fig. 4:**
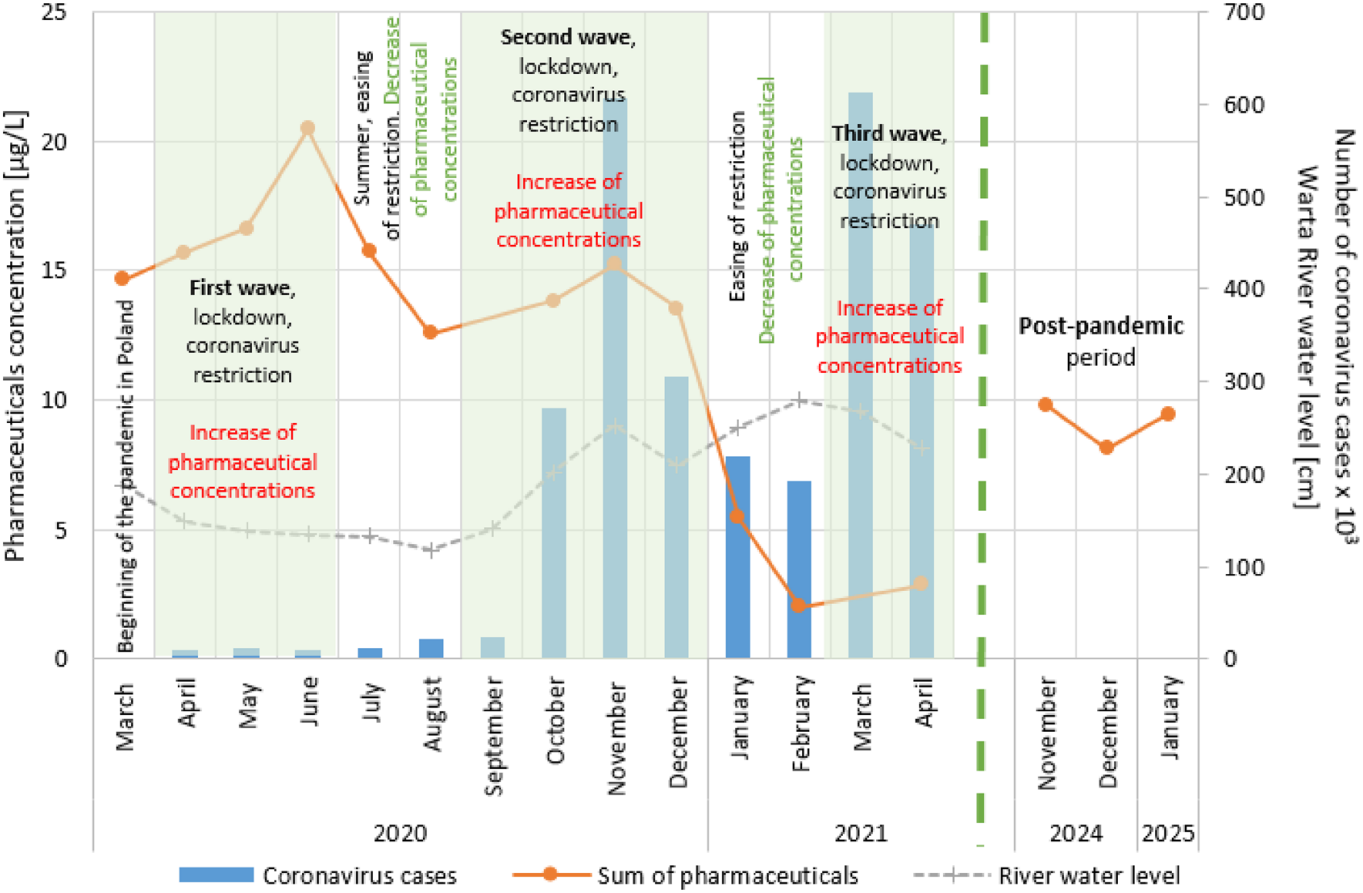
Relationship between the value of pharmaceutical concentrations and the COVID-19 pandemic and post-pandemic periods at Sampling point 2.

The variability in pharmaceutical concentrations over time reflected the stages of introducing restrictions related to the COVID-19 pandemic in Poland (Fig. 4). With the successive waves of the coronavirus, which were associated with the introduction of restrictions, the concentration of pharmaceuticals at Sampling point 2 increased. During the period of loosening restrictions, a decrease in the concentration of pharmaceuticals at Sampling point 2 was observed. One-way ANOVA indicated significant differences between the COVID-19 wave and nonwave (ease of restrictions) periods in terms of pharmaceutical concentrations. The p-value for concentration*wave/nonwave was 0.001601.

In the initial stage of the pandemic, tight restrictions were imposed despite the relatively low number of cases of coronavirus infections compared with the number at the end of the year. In the next stage, restrictions were loosened in the summer, then strengthened in autumn, loosened again in early winter and reinforced in spring.

A comparison of pharmaceutical concentrations with river water levels and precipitation during the study period, did not clearly explain the changes in pharmaceutical concentrations over time (Fig. 4; Table 1). Fluctuations in water level did not reflect fluctuations in pharmaceutical concentrations because these values mostly they do not coincide with each other. The Spearman correlation for concentration*water level was − 0.3846 (weak correlation), and that for concentrations*precipitations was − 0.2657 (weak correlation).

**Fig. 5:**
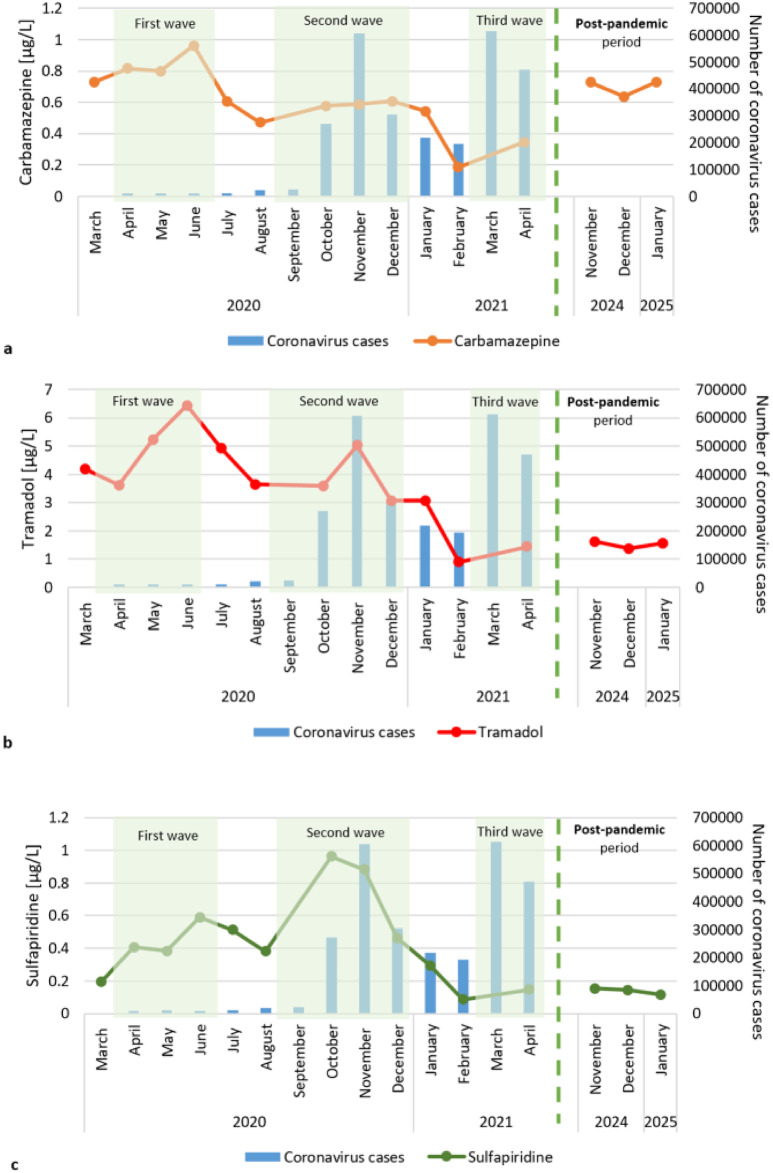

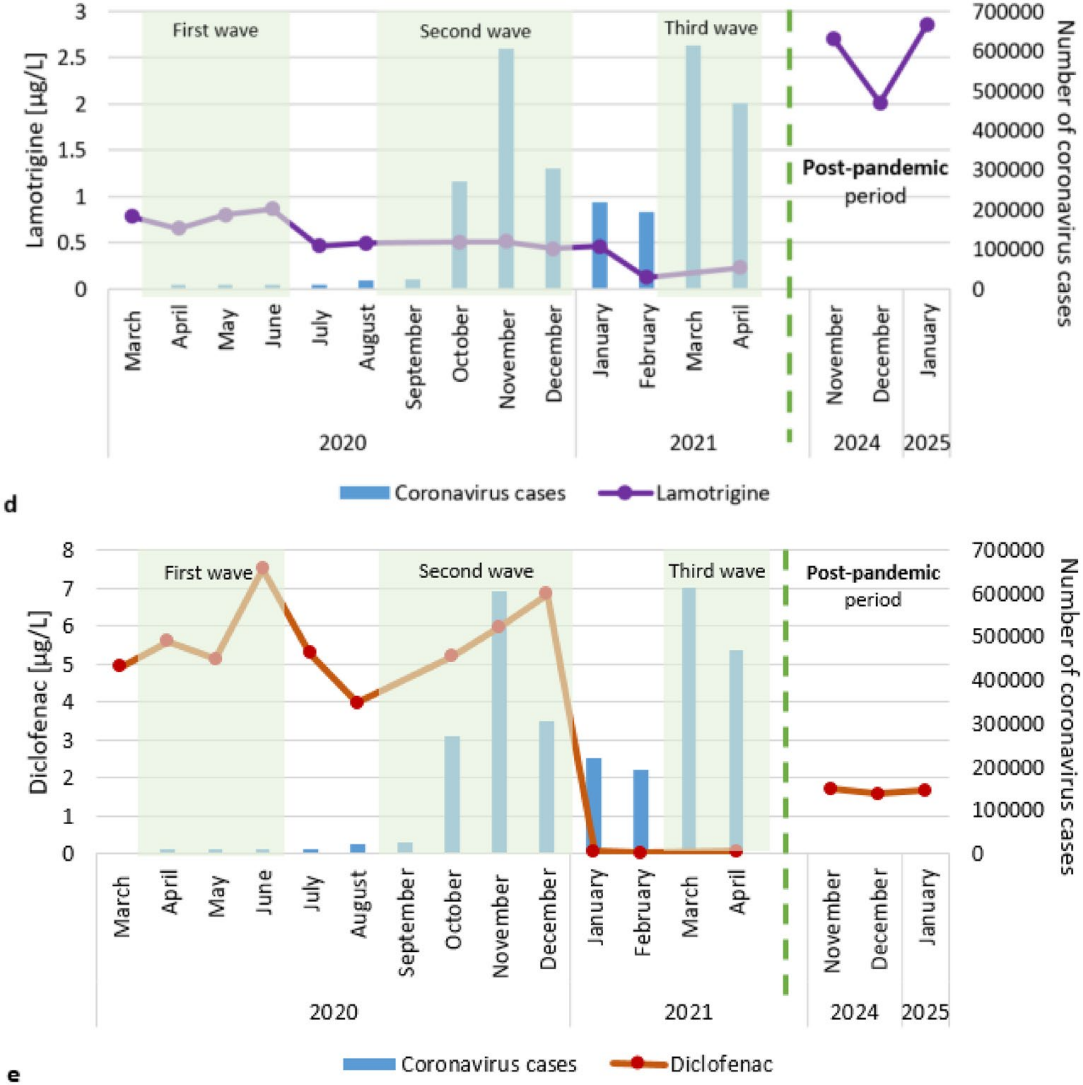
The concentrations of pharmaceuticals in river water near the treated sewage outflow point increased during successive waves of the pandemic.

The trend of increasing concentrations during subsequent waves included carbamazepine, (tramadol, sulfapiridine, diclofenac and lamotrigine (Fig. 5). The highest concentration of these pharmaceuticals was recorded in the first wave of the pandemic and was subsequently lower during the following waves. The exception was sulfapiridine, which reached the highest concentration during the second wave.

**Fig. 6:**
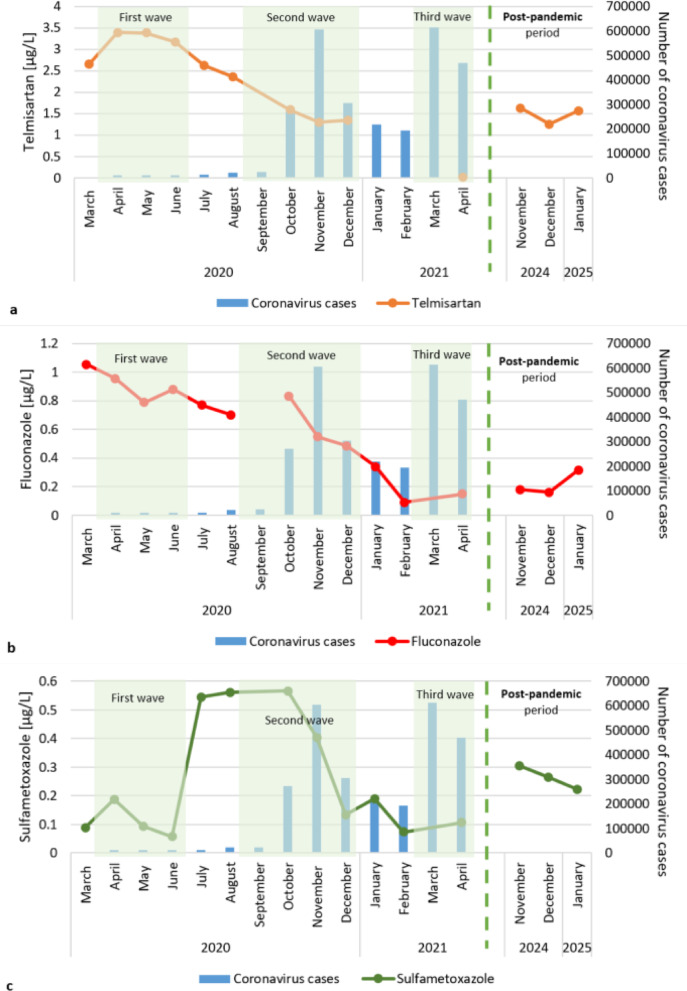
Pharmaceutical concentrations decrease in river water near treated sewage during successive waves of the pandemic.

In contrast, some pharmaceuticals decreased in concentration in the Warta River near the treated sewage outflow point during the entire study period (Fig. 6). The highest concentrations were recorded in March 2020 at the start of the coronavirus pandemic in Poland, followed by lower concentrations. Among these substances, telmisartan and fluconazole can be distinguished. Sulfamethoxazole exhibited a different trend. Sulfamethoxazole concentrations decreased during the first wave of the pandemic, increased in June 2020, and then began to decrease again in November 2020.

**Fig. 7:**
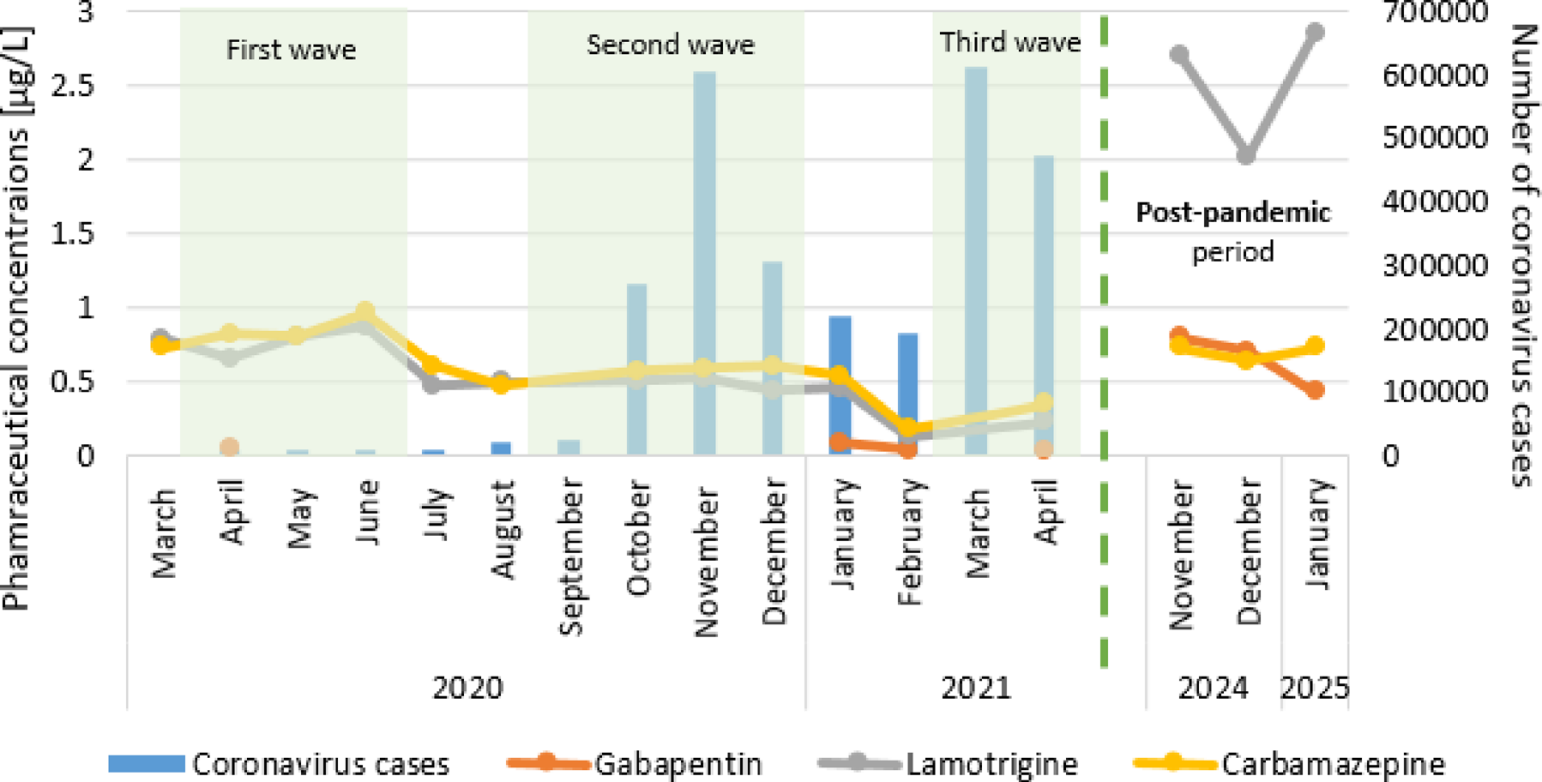
Pharmaceuticals concentrations increased in river water near the treated sewage during the post-pandemic period.

In the post-pandemic period, there was an increase in the use of pharmaceuticals such as psychotropic drugs, mood stabilizers and anti-anxiety drugs (lamotrigine, carbamazepine and gabapentin) (Fig. 7). The lamotrigine concentrations during the pandemic period range from 0.13 to 0.87 µg/L. During the post-pandemic period, it increased to 2.02–2.85 µg/L. During the pandemic, gabapentin was detected only 4 times at levels above the limit of quantification, reaching a maximum concentration of 0.09 µg/L. In contrast during the postpandemic period, it was detected in each sampling campaign at concentrations of 0.43–0.80 µg/L. The concentrations of carbamazepine, a psychotropic, anticonvulsant and mood stabilizing drug, remained high (0.64–0.73 µg/L) during the post-pandemic period.

Average concentrations of sulfamethoxazole (0.23–0.31 µg/L) and telmisartan (1.26–1.62 µg/L) were recorded during the post-pandemic period in relation to the pandemic period (Figs. 5 and 6). However, diclofenac, fluconazole, tramadol and sulfapiridine occurred at concentrations comparable to the lower values detected during the pandemic (Figs. 5 and 6).

## Discussion

The COVID-19 pandemic has significantly changed the world. It affected humans (health, work, education, and social life) and the environment (climate, water, and air)^31–33^. Changes in surface water quality are also visible. One type of river water pollution strongly dependent on the pandemic is pharmaceuticals, which enter rivers through the discharge of treated sewage^43,47^. Sewage treatment plants are among the main sources of pharmaceuticals in the environment^18,67,68^. Research conducted in the Warta River (Poland) upstream and downstream of the sewage treatment plant during the COVID-19 pandemic and in the post-pandemic period allowed to confirm this statement. Higher concentrations of pollutants were observed near the outflow point of the treated sewage, and the lowest concentrations were observed in places not exposed to the discharge from the sewage treatment plant. Most sewage treatment plants are not designed to effectively remove pharmaceuticals, and the facility investigated in our study is an example of such a case^69^. The COVID-19 pandemic period, which resulted in a notable increase in pharmaceutical consumption, has made the problem even more serious^43,44,70,71^. However, it should be emphasized that pharmaceutical concentrations are significantly reduced within 500 m of the outflow point of the treated sewage. Dilution may be considered the main reason for the decrease in pharmaceutical concentrations in such a short section of the river. At the treated sewage outflow point, the share of surface water is much lower than that at the next sampling point, which is 500 m away. The existence of statistically significant differences between the sampling points was confirmed by the ANOVA test concentration*sampling points (p-value = 1.51E-09). The test was adapted from the method described by Niemi et al. 2022^72^.

The above mentioned increase in pharmaceutical consumption during the COVID-19 pandemic should theoretically be reflected in higher concentrations of pollutants in sewage. However, the research conducted in Warta River water near the sewage treatment outflow has shown that the expected increase in the pharmaceutical concentration is not clear. The pandemic period should be divided into two distinct phases: the time of lockdown (the COVID-19 wave) and the period of easing of restrictions. Human activity significantly influences pharmaceutical concentrations in the environment; therefore, analyzing the COVID-19 pandemic as a single period will not yield accurate conclusions. The introduction of subsequent lockdowns and easing of restrictions constantly changed people’s activities, which was visible in the concentrations of pharmaceuticals (Fig. 4). It is essential to consider the varying levels of human activity during different pandemic phases to assess their impact on pharmaceutical contamination in the environment. Additionally, pharmaceuticals should be analyzed on a single substance basis, because they show different trends in their concentrations and behavior, depending on use purpose, treated diseases, and availability (OTC/prescription medicine). It is important to acknowledge certain limitations of the study. These data were obtained from 15 research campaigns covering the collection of single samples from three sampling points, including river water near the outflow point of the treated sewage. The system is extremely complex, combining environmental factors, as well as those related to the consumptions of pharmaceuticals and their removal in the sewage treatment plant.

The concentration of pharmaceuticals at Sampling point 2 (river water near treated sewage discharge) increased mainly during subsequent waves of the COVID-19 pandemic, that were associated with the implementation of lockdowns and additional restrictions. This observation confirms the results of the ANOVA test, which revealed significant differences between the analyzed periods (p-value = 0.001601). In each of the three analyzed waves, the sum of the pharmaceutical concentrations increased, although the increase was smaller with each lockdown (Fig. 4). The sum of pharmaceutical concentrations was the highest in the first wave of the pandemic, despite the lowest number of coronavirus cases. In contrast, the concentration was lowest in the third wave, despite the high incidence of COVID-19. The observed increase in the pharmaceutical concentrations in the river water during the subsequent waves of the COVID-19 was potentially caused by the treatment of an increased number of infections as well as prevention, the desire to protect against the virus and fear^73,74^. The lower total sum of pharmaceutical concentrations during each subsequent lockdown was due to the closure of some hospitals and health clinics, and the reduction in nonurgent medical consultation, which made treatment and continuing consumption of prescription medicines difficult^49,75,76^. The significant decrease in pharmaceutical consumption in the third wave may have also been influenced by the introduction of mass vaccination in 2021^77^. The general decrease in pharmaceutical concentrations during the study period may also be related to the increase in river water level (Fig. 4; Table 1). Larger volumes of uncontaminated water could have caused the dilution of contaminants from the sewage treatment plant^78^.

During periods of easing restrictions (between lockdowns), the concentration of pharmaceuticals decreased (Fig. 4). There are certainly many factors that contribute to this pharmaceutical behavior. This cannot be clearly explained by changing river levels, because fluctuations in river water levels do not coincide with the variability in pharmaceutical concentrations (Spearman’s correlation coefficient of -0.3846, weak correlation). Additionally, no statistically significant relationship was detected between precipitation and pharmaceutical concentrations. The impact of the COVID-19 pandemic is therefore the most likely explanation. During the easing of restrictions, the reduction in pharmaceutical consumption which be explained not only by a decreased demand for medical substances due to fewer illnesses but also to a decline in preventive measures caused by a sense of reduced risk. Subsequent waves of the pandemic and the accompanying restrictions were associated with increased medication use, due to the increase in the number of patients and heightened anxiety^54^. The periods of easing restrictions provided a moment of respite for people fatigued by pandemic-related restrictions, offering them a temporary sense of freedom.

A report on the behavior of doctors and patients during the COVID-19 pandemic and the distributions of medicines in 2020 indicated that there were decreases in illnesses related to infection, allergies, and muscle and joint diseases^79^. However, the largest increases were related to the treatment of patients with depression, epilepsy and schizophrenia. The number of prescriptions issued decreased by 8%, but significant differences were noted across pharmaceutical groups. For infectious diseases and prescriptions issued by pediatricians and otolaryngologists, the number decreased by approximately 30%. In contrast, prescriptions issued by psychiatrists increased (+ 3%).

The concentrations of single pharmaceuticals confirmed the abovementioned dependence. During subsequent waves of the pandemic, increase in the concentrations of analgesics (tramadol, diclofenac), antibacterial drugs (sulfapiridine), and psychotropic drugs responsible for mood stabilization (carbamazepine, lamotrigine) were observed in the Warta River water. Tramadol is an opioid pain reliever, and diclofenac is an anti-inflammatory, analgesic and antipyretic drug. In April 2020, there was an increase in pain-relieving pharmaceuticals to meet the COVID-19 treatment needs^52,80^. An increase in the use of analgesic drugs (diclofenac) during the COVID-19 pandemic was noted in Ghana’s surface water^50^ and in the Msunduzi and Umgeni rivers in KwaZulu-Natal Province of South Africa^81^. Similar results (increases in ibuprofen, anti-inflammatory, analgesic and antipyretic drugs) were obtained in studies, where the pharmaceutical concentrations in the Warta River (upstream of the sewage treatment plant) were compared across three periods (2012, 2013/2014 and 2019/2020)^49^. A study conducted in Greece revealed a minor increase in antibiotics use during the increased number of infections^54^. An increase in the number of patients, a prolonged pandemic, a decrease in living standards, and a lack of access to entertainment could cause a decrease in well-being, leading to greater consumption of psychotropic drugs^82^. Rogers et al.^83^ reported that poor mental health related to the pandemic was associated with the start of the use of psychoactive substances. Studies conducted in the United States confirmed an increase in the incidence of depression during the pandemic^84^. Similar to the results of the present research in the Warta River, an increase in the concentration of psychotropic drugs during the COVID-19 pandemic was observed in sewage samples from Connecticut, USA^52^. In that research, no trend was noted for tramadol or carbamazepine. However, these studies cover a short period of time (March-June 2020), while the present study in the Warta River covered 15 sampling campaigns, when there were as many as three lockdowns and periods of easing restrictions. In Croatia (Drava and Sava River) a decrease in the use of antidepressant drugs was observed, and the authors attributed the finding to fewer stressful situations related to limited interpersonal interactions^53^. Different trends in the use of psychotropic drugs in other countries may indicate the implementation of different approaches to address with the pandemic. Notably, other active substances in a group of psychotropic drugs were also analyzed.

Moreover, decreases in the concentrations of some pharmaceuticals in river water at treated sewage outflow point have been noted during successive waves of the pandemic, including telmisartan (an antihypertensive drug), fluconazole (an antifungal drug) and sulfamethoxazole (a bacteriostatic sulphonamide antibiotic). A similar trend is noted for diuretics and cardiological drugs observed in major rivers in Croatia. Their concentrations decreased at the beginning of the pandemic and then increased after the main waves^53^. The concentration of sulphonamide antibiotics (sulfamerazine) in Lake Chenhu water was lower during the COVID-19 pandemic than before in 2019^51^. Studies of sulfamethoxazole conducted in Ecuador have shown similar results. The level of sulfamethoxazole used during the pandemic was below the limit of quantification^34^. Importantly, these are prescription pharmaceuticals, so the decline in these substances may be related to their discontinuation due to limited access to doctors. This may also result from not renewing the prescriptions due to nonattendance at a health center. In the sewage sample from the state of Connecticut, a decrease in levorphanol, an opioid used as a preoperative drug, was observed, potentially due to the reduction in elective procedures during the study period^52^. Because of fear of coronavirus infection, people, especially seniors, avoided places with a relatively high risk of coronavirus transmission.

The total pharmaceutical concentrations in river water near the treated sewage outflow point during the post-pandemic period was at an average level compared with that during the pandemic period. The concentrations are lower than those in the first and second waves of the pandemic but higher than those during the third wave. These post-pandemic pharmaceutical concentrations are most similar to the values from the period of easing restrictions. The post-pandemic period and periods of easing restrictions can be characterized as times of similar human behaviors related to the reduction in the fear of illness. Both phases likely experienced normalization of social and health care activities, resulting in similar patterns of pharmaceutical consumption. This confirms previous results showing that lockdowns had the greatest impact on the increase in human consumption of pharmaceuticals. Similar conclusions regarding antibiotics were obtained by Petromelidou et al.^54^. However, there are few studies available that include both pandemic and post-pandemic periods for the same location and the same substances tested.

When individual pharmaceuticals detected in river water during the post-pandemic period were analyzed, significant increases in the concentrations of lamotrigine (an antiepileptic drug and a mood stabilizing drug) and gabapentin (an antiepileptic and antianxiety drug) were observed compared with those during the pandemic period. During the pandemic, gabapentin was detected at levels above the LOQ in the river only 4 times. However, during the post-pandemic period, gabapentin was detected at high concentrations in each sampling campaign. Carbamazepine, which is also a psychotropic pharmaceutical and mood stabilizer, was also present at higher concentrations than it was during the pandemic. Higher concentrations of these pharmaceuticals in the post-pandemic period may reflect a general deterioration in the mental health of the population, potentially linked to the prolonged psychological impacts of the pandemic^84^. This is confirmed by data collected from inpatients at Beijing Huilongguan Hospital during the post-pandemic period, indicating an increase in the consumption of psychotropic drugs^85^. The dispensation of antipsychotics and antidepressants was greater in the post-pandemic period than before in Italy^86^ and Estonia^87^. Another pharmaceutical in the Warta River, sulfamethoxazole (a bacteriostatic sulfonamide antibiotic), typically presented higher concentrations in the post-pandemic period than in the pandemic period (except 4 sampling campaigns). Sulfamethoxazole concentrations were higher than those observed during the first and third waves, but lower than those recorded during the second wave. Higher concentrations were mostly observed because the post-pandemic period was characterized as a time of return to the normal functioning of health centers and easier access to doctors, which could have led to more frequent diagnosis and treatment of bacterial infections, which were postponed or untreated during the pandemic.

Research has shown lower concentrations of antifungal (fluconazole) and antibacterial drugs (sulfapiridine) in rivers during the post-pandemic period. The lower concentrations of these substances in the environment suggest lower consumption of these substances, which may be related to the reduction in the number of infections after the COVID-19 pandemic. Low concentrations of tramadol were also recorded after the pandemic. Tramadol is a strong analgesic and opioid used to manage generalized pain, including pain associated with COVID-19, as well as chronic and acute pain. During the pandemic, its use was particularly prevalent, as patients faced difficulties accessing medical appointments and receiving standard treatments for their disease^75,88^. With the normalization of health care services, patients regain access to standard treatments and regular care, leading to a reduction in their reliance on opioids such as tramadol.

Telmisartan is a pharmaceutical, whose concentrations in the post-pandemic period were approximately half of those observed during the pandemic. Research conducted by Daoud and Ronen^75^ indicates that during the pandemic the number of visits to cardiologists decreased by 50%. The avoidance of health centers and limited access to health care during the pandemic may have led to the discontinuation of this medication, with some patients not resuming its use after the pandemic^88^. Additionally, the pandemic heightened public awareness of health care and lifestyle modifications, which may have contributed to improved hypertension control and a reduced need for medications such as telmisartan.

## Conclusions

In the presented research, the impact of the COVID-19 pandemic on pharmaceutical concentrations in river water was analyzed, with varying trends observed during different phases of the pandemic (lockdowns and ease of restrictions). An attempt was made to assess the social condition of society on the type and amount of pharmaceuticals entering river with discharges of treated sewage and available information about the COVID-19 pandemic. As the research was performed in a river influenced by treated effluents, it is important to recognize certain limitations resulting from the complexity of the system, such as environmental conditions, WWTP operation and pharmaceutical consumption and behavior.

The pharmaceutical concentrations in the Warta River water were influenced by changes in human activity and behavior during lockdowns and periods of easing restrictions. The highest concentrations were observed during lockdowns, reflecting an increase in the use of pharmaceuticals due to increased infection rates, changes in health care access and heightened anxiety. However, the concentrations decreased during periods of easing restrictions, likely because of a reduced demand for medical substances, resulting from fewer illnesses and a decline in preventive measures caused by a sense of reduced risk.

The concentrations of individual pharmaceuticals, such as analgesics (diclofenac, tramadol), psychotropic drugs (carbamazepine, lamotrigine), and an antibacterial drug (sulfapiridine) increased during subsequent waves of the pandemic. Increases in these substances may be related to the psychological and physical impact of the pandemic on people, including pain treatment and mental health problems. The concentrations of an antihypertensive drug (telmisartan), an antifungal drug (fluconazole) and a bacteriostatic sulphonamide antibiotic (sulfamethoxazole) decreased during subsequent lockdowns. This trend may be due to limited access to medical services and prescription renewals during pandemic restrictions.

In the post-pandemic period, the total pharmaceutical concentrations were similar to those observed during the period of easing restrictions (between lockdowns). These two periods may be connected with similar patterns of human activity and more typical pharmaceutical consumption patterns. However, pharmaceutical concentrations, such as those of lamotrigine and gabapentin, have increased significantly, likely reflecting long-term mental health impacts.

The above conclusions were supported by statistical analysis (ANOVA test), which revealed significant differences between the sampling points, as well as between the periods of lockdown and the easing of restrictions.

Studies have indicated that analyzing data from the COVID-19 pandemic should be divided into two phases–lockdowns and easing of restrictions. However, this is typically not practiced in the literature. Environmental data, such as river water quality near treated sewage outflow point, can be used to describe the state of society. However, to capture the seasonal variations effectively and provide an accurate representation, the research should be long-term (at least 12 months). Moreover, research should be performed near the treated sewage outflow point to avoid data distortion by external factors.

These findings confirm that sewage treatment plants are the main source of pharmaceuticals in the environment. This finding indicates the need to improve sewage treatment technologies to reduce the release of pharmaceuticals into the ecosystem. Additionally, it highlights the importance of raising public awareness about the environmental impact of pharmaceutical consumption. This is particularly crucial for the Warta River, as it is a source of water for multiple riverbank filtration well fields that supply drinking water to the population.

## Supplementary Information

Below is the link to the electronic supplementary material.

Supplementary Material 1

## Data Availability

All data generated or analysed during this study are included in this published article.

## References

[1] Chen, M. et al. Micropollutants but high risks: human multiple stressors increase risks of freshwater ecosystems at the megacity-scale. *J. Hazard. Mater.* **460**, 132497. 10.1016/j.jhazmat.2023.132497 (2023). 37688870 10.1016/j.jhazmat.2023.132497

[2] Ren, H., Troger, R., Ahrens, L., Wiberg, K. & Yin, D. Screening of organic micropollutants in Raw and drinking water in the Yaggtze river delta, China. *Environ. Sci. Europe*. **32**, 67. 10.1186/s12302-020-00342-5 (2020).

[3] Kumar, V. et al. Global evaluation of heavy metal content in surface water bodies: A meta-analysis using heavy metal pollution indices and multivariate statistical analyses. *Chemosphere* **236**, 124364. 10.1016/j.chemosphere.2019.124364 (2019). 31326755 10.1016/j.chemosphere.2019.124364

[4] Dragon, K., Drożdżyński, D., Górski, J. & Kruć, R. The migration of pesticide residues in groundwater at a bank filtration site (Krajkowo well field, Poland). *Environ. Earth Sci.* **78**, 593. 10.1007/s12665-019-8598-0 (2019).

[5] Elfikrie, N., Ho, Y. B., Zaidon, S. Z., Juahir, H. & Tan, E. S. S. Occurrence of pesticides in surface water, pesticides removal efficiency in drinking water treatment plant and potential health risk to consumers in Tengi river basin, Malaysia. *Sci. Total Environ.* **712**, 136540. 10.1016/j.scitotenv.2020.136540 (2020). 32050383 10.1016/j.scitotenv.2020.136540

[6] Kruć-Fijałkowska, R., Dragon, K., Drożdżyński, D. & Górski, J. Seasonal variation of pesticides in surface water and drinking water wells in the annual cycle in Western poland, and potential health risk assessment. *Sci. Rep.* **12**, 1. 10.1038/s41598-022-07385-z (2022). 35228621 10.1038/s41598-022-07385-z PMC8885637

[7] Dragon, K., Górski, J., Kruć, R., Drożdżyński, D. & Grischek, T. Removal of natural organic matter and organic micropollutants during riverbank filtration in krajkowo, Poland. *Water* **10**, 1457. 10.3390/w10101457 (2018).

[8] Kruć-Fijałkowska, R., Dragon, K. & Drożdżyński, D. Factors affecting the concentrations of pharmaceutical compounds in river and groundwaters: efficiency of river bank filtration (Mosina-Krajkowo well field, Poland). *Geol. Q.* **66**, 3. 10.7306/gq.1635 (2022).

[9] Li, W. C. Occurrence, sources, and fate of pharmaceuticals in aquatic environment and soil. *Environ. Pollut.* **187**, 193–201. 10.1016/j.envpol.2014.01.015 (2014). 24521932 10.1016/j.envpol.2014.01.015

[10] Huang, Q., Liu, M., Cao, X. & Liu, Z. Occurrence of microplastics pollution in the Yangtze river: distinct characteristics of Spatial distribution and basin-wide ecological risk assessment. *Water Res.* **229**, 119431. 10.1016/j.watres.2022.119431 (2023). 36527870 10.1016/j.watres.2022.119431

[11] Nikolaou, A. D., Sureyya, M. & Fatta, D. Occurrence patterns of pharmaceuticals in water and wastewater environments. *Anal. Bioanal Chem.* **387**, 1225–1234. 10.1007/s00216-006-1035-8 (2007). 17205270 10.1007/s00216-006-1035-8

[12] Alder, A. C., Schaffner, C., Majewsky, M., Klasmeier, L. & Fenner, K. Fate of β-blocker human pharmaceuticals in surface water: comparison of measured and simulated concentrations in the Glatt Valley watershed, Switzerland. *Water Res.* **44**, 3, 936–948. 10.1016/j.watres.2009.10.002 (2010). 19889439 10.1016/j.watres.2009.10.002

[13] Bandala, E. R. et al. Impacts of COVID-19 pandemic on the wastewater pathway into surface water: a review. *Sci. Total Environ.* **774**, 145586. 10.1016/j.scitotenv.2021.145586 (2021). 33607440 10.1016/j.scitotenv.2021.145586 PMC7862925

[14] Macedo, H. E. et al. Distribution and characteristics of wastewater treatment plants within the global river network. *Earth Syst. Sci. Data*. **14**, 559–577. 10.5194/essd-14-559-2022 (2022).

[15] Matesun, J., Petrik, L., Musvoto, E., Ayinde, W. & Ikumi, D. Limitations of wastewater treatment plants in removing trace antropogenic biomarkers and future directions: A review. *Ecotoxicol. Environ. Saf.* **281**, 116610. 10.1016/j.ecoenv.2024.116610 (2024). 38909392 10.1016/j.ecoenv.2024.116610

[16] Dai, H., Wang, C., Yu, W. & Han, J. Tracing COVID-19 drugs in the environment: are we focusing on the right environmental compartment? *Environ. Pollut.* **339**, 122732. 10.1016/j.envpol.2023.122732 (2023). 37838316 10.1016/j.envpol.2023.122732

[17] Sui, Q. et al. Occurrence, sources and fate of pharmaceuticals and personal care products in the ground water: a review. *Emerg. Contaminants*. **1**, 14–24. 10.1016/j.emcon.2015.07.001 (2015).

[18] Ślósarczyk, K., Jakóbczyk-Karpierz, S., Różkowski, J. & Witkowski, A. J. Occurrence of pharmaceuticals and personal care products in the water environment of poland: a review. *Water* **13**, 2283. 10.3390/w13162283 (2021).

[19] Kasprzyk-Hordern, B., Dinsdale, R. M. & Guwy, A. J. The removal of pharmaceuticals, personal care products, endocrine disruptors and illicit drugs during wastewater treatment and its impact on the quality of receiving waters. *Water Res.* **43**, 2, 363–380. 10.1016/j.watres.2008.10.047 (2009). 19022470 10.1016/j.watres.2008.10.047

[20] Styszko, K., Proctor, K., Castrignano, E. & Kasprzyk-Hordern, B. Occurrence of pharmaceutical residues, personal care products, lifestyle chemicals, illicit drugs and metabolites in wastewater and receiving surface waters of Krakow agglomeration in South Poland. *Sci. Total Environ.* **768**, 144360. 10.1016/j.scitotenv.2020.144360 (2021). 33450690 10.1016/j.scitotenv.2020.144360

[21] Chaturvedi, K., Vishwakarma, D. K. & Singh, N. COVID-19 and its impact on education, social life and mental health of students: A survey. *Child. Youth Serv. Rev.* **25**, 121, 105866. 10.1016/j.childyouth.2020.105866 (2021). 10.1016/j.childyouth.2020.105866 PMC7762625 33390636

[22] Hosseinzadeh, P., Zareipour, M., Baljani, E. & Moradali, M. R. Social consequences of the COVID-19 pandemic. A systematic review. *Invest. Educ. Enferm*. **30, 40**, (1), e10. 10.17533/udea.iee.v40n1e10 (2022). 10.17533/udea.iee.v40n1e10 PMC9052715 35485623

[23] Ventriglio, A., Castaldelli-Maia, J. M., Torales, J., Chumakov, E. M. & Bhugra, D. Personal and social changes in the time of COVID-19. *Ir. J. Psychol. Med.* **9**, 1–3. 10.1017/ipm.2021.23 (2021). 10.1017/ipm.2021.23 PMC8060536 33685538

[24] Ilnicka, E., Kasprzyk, D., Ogórek, A., Sternak, A. & Cierpka, A. The COVID-19 pandemic in the experience of people in late adulthood. *Psychologia Rozwojowa (in polish)*. **26**, 2, 59–77. 10.4467/20843879PR.21.012.15135 (2021).

[25] Roy, A., Deb, S. & Chakarwarti, D. Impact of COVID-19 on public social life and mental health: a statistical study of Google trends data from the USA. *J. Appl. Stat.* **51** (3), 581–605. 10.1080/02664763.2022.2164562 (2023). 38370267 10.1080/02664763.2022.2164562 PMC10868428

[26] Zhang, J. et al. The differential psychological distress of populations affected by the COVID-19 pandemic. *Brain Behav. Immun.* **87**, 49–50. 10.1016/j.bbi.2020.04.031 (2020). (2020). 32304883 10.1016/j.bbi.2020.04.031 PMC7156946

[27] Moccia, L. et al. Affective temperament, attachment style, and the psychological impact of the COVID-19 outbreak: an early report on the Italian general population. *Brain Behav. Immun.* **87**, 75–79. 10.1016/j.bbi.2020.04.048 (2020). 32325098 10.1016/j.bbi.2020.04.048 PMC7169930

[28] Probst, T., Stippl, P. & Pieh, C. Changes in provision of psychotherapy in the early weeks of the COVID-19 lockdown in Austria. *Int. J. Environ. Res. Public. Health*. **17**, 11, 3815. 10.3390/ijerph17113815 (2020). 32471295 10.3390/ijerph17113815 PMC7312759

[29] Ang, L., Hernandez-Rodriguez, E., Cyriaque, V. & Yin, X. COVID-19’s environmental impacts: challenges and implications for the future. *Sci. Total Environ.* **899**, 165581. 10.1016/j.scitotenv.2023.165581 (2023). 37482347 10.1016/j.scitotenv.2023.165581

[30] Diffenbaugh, N. S. COVID-19 and the environment: Short-run and potential long-run impacts. *Annu. Rev. Environ. Resour.* **47**, 65–90. 10.1146/annurev-environ-120920-125207 (2022).

[31] Bhat, S. A. et al. Impact of COVID-related lockdowns on environmental and climate change scenarios. *Environ. Res.* **195**, 110839. 10.1016/j.envres.2021.110839 (2021). 33549623 10.1016/j.envres.2021.110839 PMC7860963

[32] Raza, T. et al. Impact assessment of COVID-19 global pandemic on water, environment, and humans. *Environ. Adv.* **11**, 100328. 10.1016/j.envadv.2022.100328 (2023). 36532331 10.1016/j.envadv.2022.100328 PMC9741497

[33] Uddin, M. G. et al. Assessing the impact of COVID-19 lockdown on surface water quality in Ireland using advanced Irish water quality index (IEWQI) model. *Environ. Pollut*. **336**, 122456. 10.1016/j.envpol.2023.122456 (2023). 37673321 10.1016/j.envpol.2023.122456

[34] Cipriani-Avila et al. Occurrence of emerging contaminants in surface water bodies of a coastal Province in Ecuador and possible influence of tourism decline caused by COVID-19 lockdown. *Sci. Total Environ.* **866**, 161340. 10.1016/j.scitotenv.2022.161340 (2023). 36603613 10.1016/j.scitotenv.2022.161340 PMC9807265

[35] Ormaza-González, F., Castro-Rodas, D. & Statham, P. COVID-19 impacts on beaches and coastal water pollution at selected sites in ecuador, and management proposals post-pandemic. *Front. Mar. Sci.* **8**, 669374. 10.3389/fmars.2021.669374 (2021).

[36] Zambrano-Monserrate, M. A., Ruano, M. & Sanchez-Alcalde, L. Indirect effects of COVID-19 on the environment. *Sci. Total Environ.* **728**, 138813. 10.1016/j.scitotenv.2020.138813 (2020). 32334159 10.1016/j.scitotenv.2020.138813 PMC7169883

[37] Luczkiewicz, A. et al. Wastewater quality during the COVID-19 pandemic: a retrospective analysis of Polish case study. *Int. J. Environemntal Sci. Technol.* **22**, 4125–4142. 10.1007/s13762-024-05934-9 (2025).

[38] Tokatli, C. & Varol, M. Impact of the COVID-19 lockdown period on surface water quality in the Meriç-Ergene river basin, Northwest Turkey. *Environ. Res.* **111051**10.1016/j.envres.2021.111051 (2021). 10.1016/j.envres.2021.111051 33753075

[39] Chakraborty, B. et al. Positive effects of COVID-19 lockdown on river water quality: evidence from river damodar, India. *Sci. Rep.* **11**, 20140. 10.1038/s41598-021-99689-9 (2021). 34635728 10.1038/s41598-021-99689-9 PMC8505400

[40] Qiao, X. et al. Surface water quality in the upstream-most megacity of the Yangtze river basin (Chengdu): 2000–2019 trends, the COVID-19 lockdown effects, and water governance implications. *Environ. Sustain. Indic.* **10**, 100118. 10.1016/j.indic.2021.100118 (2021).

[41] Singh, M., Pandey, U. & Pandey, J. Effects of COVID-19 lockdown on water quality, microbial extracellular enzyme activity, and sediment-P release in the Ganga river, India. *Environ. Sci. Pollut Control Ser.* **29**, 60968–60986. 10.1007/s11356-022-20243-9 (2022). 10.1007/s11356-022-20243-9 PMC9014407 35435553

[42] Yunus, A. P., Masago, Y. & Hijioka, Y. COVID-19 and surface water quality: improved lake water quality during the lockdown. *Sci. Total Environ.* **731**, 139012. 10.1016/j.scitotenv.2020.139012 (2020). 32388159 10.1016/j.scitotenv.2020.139012 PMC7185006

[43] Gwenzi, W. et al. COVID-19 drugs in aquatic systems: a review. *Environ. Chem. Lett.* **20**, 1275–1294. 10.1007/s10311-021-01356-y (2022). 35069060 10.1007/s10311-021-01356-y PMC8760103

[44] Love, J. S., Blumenberg, A. & Horowitz, Z. The parallel pandemic: medical misinformation and COVID-19: *Primum Non nocere*. *J. Gen. Intern. Med.* **35**, 2435–2436. 10.1007/s11606-020-05897-w (2020). 32410129 10.1007/s11606-020-05897-w PMC7224586

[45] Olesch, A. et al. *Pol. Health J.* (2022). https://issuu.com/polishhealthcarejournal/docs/01_2022_osoz?utm_medium=referral&utm_source=blog.osoz.pl

[46] Diaz-Calam, N. et al. Consumption and occurrence of antidepressants (SSRIs) in pre- and post-COVID-19 pandemic, their environmental impact and innovative removal methods: A review. *Sci. Total Environ.* **829**, 154656. 10.1016/j.scitotenv.2022.154656 (2022). 35318057 10.1016/j.scitotenv.2022.154656

[47] Pashaei, R. et al. Pharmaceutical and microplastic pollution before and during the COVID-19 pandemic in surface water, wastewater, and groundwater. *Water* **14**, 3082. 10.3390/w14193082 (2022).

[48] Wojcieszyńska, D., Guzik, H. & Guzik, U. Non-steroidal anti-inflammatory drugs in the era of the COVID-19 pandemic in the context of the human and the environment. *Sci. Total Environ.* **834**, 155317. 10.1016/j.scitotenv.2022.155317 (2022). 35452725 10.1016/j.scitotenv.2022.155317 PMC9015952

[49] Antos, J. et al. Monitoring of contamination of the warta river in Poznan by non-steroidal anti-inflammatory drugs and antibiotics. *Water* **15**, 2716. 10.3390/w15152716 (2023).

[50] Azanu, D., Adu-Poku, D., Saah, S. A. & Appaw, W. O. Prevalence of pharmaceuticals in surface water samples in Ghana. *J. Chem.* **477**10.1155/2021/7829477 (2021).

[51] Ma, N. et al. Distribution of antibiotics in lake water-groundwater-sediment system in Chenhu lake area. *Environ. Res.* **204**, 112343. 10.1016/j.envres.2021.112343 (2022). 34748778 10.1016/j.envres.2021.112343

[52] Nason, S. L. et al. Changes in sewage sludge chemical signatures during a COVID-19 community lockdown, part 1: traffic, drugs, mental health, and disinfectants. *Environ. Toxicol. Chem.* **41**, 1179–1192. 10.1002/etc.5217 (2022). 34668219 10.1002/etc.5217 PMC8653241

[53] Stipanicev, D. et al. COVID-19 lockdowns—Effect on concentration of pharmaceuticals and illicit drugs in two major Croatian rivers. *Toxics* **10**, 241. 10.3390/toxics10050241 (2022). 35622654 10.3390/toxics10050241 PMC9143423

[54] Petromelidou, S. et al. Exploring patterns of antibiotics during and after COVID-19 pandemic in wastewaters of Northern greece: potential adverse effects on aquatic environment. *Sci. Total Environ.* **914**, 169832. 10.1016/j.scitotenv.2023.169832 (2024). 38190919 10.1016/j.scitotenv.2023.169832

[55] Pielach, M. & Mizerna-Nowotna, P. Lewobrzeżna Oczyszczalnia Ścieków Dla Miasta Poznania – testy Redukcji Siarkowodoru Przy Użyciu chemii – etap II. *Forum Eksploatatora*. **1**, 94, 32–34 (2018). (in Polish).

[56] Ilnicki, P., Farat, R., Górecki, K. & Lewandowski, P. Impact of Climatic change on river discharge in the driest region of Poland. *Hydrol. Sci. J.* **59** (6). 10.1080/02626667.2013.831979 (2014).

[57] Marsz, A. A., Sobkowiak, L., Styszyńska, A., Wrzesiński, D. & Perz, A. The thermal state of the North Atlantic ocean andhydrological droughts in the warta river catchment in Poland during 1951–2020. *Water* **15**, 2547. 10.3390/w15142547 (2023).

[58] R Core Team. R: A Language and Environment for Statistical Computing: The R Project for Statistical Computing. R version 4.4.3. (2025).

[59] Cucinotta, D. & Vanelli, M. WHO declares COVID-19 a pandemic. *Acta Biomed.* **91**, 1, 157–160. 10.23750/abm.v91i1.9397 (2020). 32191675 10.23750/abm.v91i1.9397 PMC7569573

[60] Ministry of Health. Report of coronavirus (SARS-CoV-2) infections (2021). https://www.gov.pl/web/koronawirus/wykaz-zarazen-koronawirusem-sars-cov-2

[61] Journal of Laws. Item 491 as amended. Regulation of the minister of health of 20 March 2020 regarding the announcement of the state of epidemic in the territory of the Republic of Poland.https://isap.sejm.gov.pl/isap.nsf/DocDetails.xsp?id=WDU20200000491 (2020).

[62] Journal of Laws. Item 1356 as amended. Regulation of the Council of ministers of 7 August 2020 on the introduction of certain restrictions, orders and prohibitions in connection with the occurrence of an epidemic state https://isap.sejm.gov.pl/isap.nsf/DocDetails.xsp?id=WDU20200001356(2020).

[63] Government Security Centre. New rules for covering the nose and mounth, open cinemas and gyms – we are entering the next stage of unfreezing (2021). https://www.gov.pl/web/koronawirus/4-etap-odmrazania

[64] Rzymski, P., Poniedziałek, B. & Fal, A. Willingness to receive the booster COVID-19 vaccine dose in Poland. *Vaccines (Basel)*. **9** (11). 10.3390/vaccines9111286 (2021). 10.3390/vaccines9111286 PMC8624071 34835217

[65] Koronawiruswpolsce.pl Statistics and data on COVID-19. in Poland. https://koronawiruswpolsce.pl. Accessed 29 April 2025.

[66] Institute of Meteorology and Water Management. – National Research Institute. https://www.danepubliczne.imgw.pl. Accessed 15 April 2025.

[67] Zhou, H. et al. Occurrence of selected pharmaceuticals and caffeine in sewage treatment plants and receiving rivers in beijing, China. *Water Environ. Res.* **82**, 11, 2239–2248. 10.2175/106143010X12681059116653 (2010). 21141385 10.2175/106143010x12681059116653

[68] Dragon, K. et al. The impact of treated wastewater effluent on contamination of a water supply aquifer during one decade of water exploitation (Tursko well field, Poland). *Geol. Q.* **66**, 14. 10.7306/gq.1646 (2022).

[69] Liu, H-Q. et al. Spatial distribution and removal performance of pharmaceuticals in municipal wastewater treatment plants in China. *Sci. Total Environ.* **586**, 1162–1169. 10.1016/j.scitotenv.2017.02.107 (2017). 28228239 10.1016/j.scitotenv.2017.02.107

[70] Manoiu, V-M., Kubiak-Wójcicka, K., Craciun, A-I., Akman, C. & Akman, E. Water quality and water pollution in time of COVID-19: positive and negative repercussions. *Water* **14**, 1124. 10.3390/w14071124 (2022).

[71] Sadio, A. J. et al. Assessment of self-medication practices in the context of the COVID-19 outbreak in Togo. *BMC Public. Health*. **21**, 58. 10.1186/s12889-020-10145-1 (2021). 33407321 10.1186/s12889-020-10145-1 PMC7787400

[72] Niemi, L. et al. Spatiotemporal trends and annual fluxes of pharmaceutical in a Scottish priority catchment. *Environ. Pollut.* **292** (A), 118295. 10.1016/j.envpol.2021.118295 (2022). 34626711 10.1016/j.envpol.2021.118295

[73] Chen, X. et al. Occurrence and risk assessment of pharmaceuticals and personal care products (PPCPs) against COVID-19 in lakes and WWTP-river-estuary system in wuhan, China. *Sci. Total Environ.* **792**, 148352. 10.1016/j.scitotenv.2021.148352 (2021). 34147798 10.1016/j.scitotenv.2021.148352 PMC8197610

[74] Usman, M., Farooq, M. & Hanna, K. Environmental side effects of the injudicious use of antimicrobials in the era of COVID-19. Sci. *Total Environ.* **747**, 141053. 10.1016/j.scitotenv.2020.141053 (2020). 10.1016/j.scitotenv.2020.141053 PMC7368658 32702547

[75] Daoud, A. & Ronen, O. Decline in emergency department visits during the COVID-19 quarantine. *Am. J. Emerg. Med.* **71**, 74–90. 10.1016/j.ajem.2023.06.002 (2023). 37352578 10.1016/j.ajem.2023.06.002 PMC10246301

[76] Mularczyk-Tomaczewska, P. et al. Barriers to accessing health services during the COVID-19 pandemic in poland: A nationwide cross-sectional survey among 109,928 adults in Poland. *Front. Public. Health*. **8**, 10986996. 10.3389/fpubh.2022.986996 (2022). 10.3389/fpubh.2022.986996 PMC9495711 36159267

[77] NIZP-PIB. Adverse post-vaccination reactions after COVID-19 vaccines in poland. Report for the period 27.12.2020–15.12.2021 (2021). https://www.pzh.gov.pl/wp-content/uploads/2021/12/Raport-NOP-do-15.12.2021.pdf

[78] Zhou, M. et al. Dilution or enrichment: the effects of flood on pollutants in urban rivers. *Environ. Sci. Europe*. **34**, 61. 10.1186/s12302-022-00639-7 (2022).

[79] IQVIA. Structure and dynamics of the pharmaceutical market, physician and patient behaviour, and drug distributions in 2020 – key facts. (2021).

[80] US Drug Enforcement Administration. DEA takes additional steps to allow increased production of controlled substances used in COVID-19 care (2020). https://www.dea.gov/press-releases/2020/04/07/dea-takes-additional-steps-allow-increased-production-controlled

[81] Omotola, E. O. & Olatunji, O. S. Quantification of selected pharmaceutical compounds in water using liquid chromatography-electrospray ionization mass spectrometry (LC-ESI-MS). *Heliyon* **6**, e05787. 10.1016/j.heliyon.2020.e05787 (2020). 33426324 10.1016/j.heliyon.2020.e05787 PMC7779709

[82] Pierce, M. et al. Mental health before and during the COVID-19 pandemic: a longitudinal probability sample survey of the UK population. *Lancet Psychiatry*. **7**, 883–892. 10.1016/S2215-0366(20)30308-4 (2020). 32707037 10.1016/S2215-0366(20)30308-4 PMC7373389

[83] Rogers, A. H., Shepherd, J. M., Garey, L. & Zvolensky, M. J. Psychological factors associated with substance use initiation during the COVID-19 pandemic. *Psychiatry Res.* **293**, 113407. 10.1016/j.psychres.2020.113407 (2020). 32827993 10.1016/j.psychres.2020.113407 PMC7434361

[84] Ettman, C. K. et al. Prevalence of depression symptoms in US adults before and during the COVID-19 pandemic. *JAMA Netw. Open.* **3**, e2019686. 10.1001/jamanetworkopen.2020.19686 (2020). 32876685 10.1001/jamanetworkopen.2020.19686 PMC7489837

[85] Yang, R. et al. Increased antipsychotic drug concentration in hospitalized patients with mental disorders following COVID-19 infection: a call for attention. *Front. Psychiatry*. **15**, 1421370. 10.3389/fpsyt.2024.1421370 (2024). 39077630 10.3389/fpsyt.2024.1421370 PMC11284031

[86] Nobili, A. et al. Post-COVID condition: dispensation of drugs and diagnostic tests as proxies of healthcare impact. *Intern. Emerg. Med.* **18**, 801–809. 10.1007/s11739-023-03228-5 (2023). 36944811 10.1007/s11739-023-03228-5 PMC10030070

[87] Mooses, K. et al. The use of prescription drugs and health care services during the 6-month post-COVID-19 period. *Sci. Rep.* **13**, 11638. 10.1038/s41598-023-38691-9 (2023). 37468497 10.1038/s41598-023-38691-9 PMC10356787

[88] Cho, Y., Yeo, I. H., Lee, D. E. & Kim, J. K. Coronavirus disease pandemic impact on emergency department visits for cardiovascular disease in korea: A review. *Medicine* **102**, e35992. 10.1097/MD.0000000000035992 (2023). 38013376 10.1097/MD.0000000000035992 PMC10681605

