# Supplementary material for: Occurrence and temporal changes of pharmaceuticals in the Warta River in Poland during and after the COVID-19 pandemic
Source: Sci Rep. 2025 Aug 12;15:29451. doi: 10.1038/s41598-025-14568-x (PMC12339683; doi:10.1038/s41598-025-14568-x)
Supplement: Supplementary file 1 — Supplementary Material 1 [file 41598_2025_14568_MOESM1_ESM.pdf]

Supplementary Table S1. Studied pharmaceuticals in the Warta River samples.

**PhACs**	**Precursor ion [*m/z*]**	**Product ion [*m/z*]**	**RT [min]**	**DP [V]**	**EP [V]**	**CE [V]**	**CXP [V]**
Carbamazepine	237.1	194.0<br>165.0	3.68	25<br>25	10<br>10	31<br>65	14<br>18
Diclofenac	296.0	213.9<br>249.8	4.26	25<br>25	10<br>10	43<br>18	11<br>19
Fluconazole	307.1	220.0<br>238.0<br>169.0	3.04	25<br>25<br>25	10<br>10<br>10	25<br>22<br>32	23<br>13<br>10
Gabapentin	172.1	154.1<br>137.1	2.44	25<br>25	10<br>10	17<br>22	10<br>7
Lamotrigine	256.0	210.9<br>165.0<br>158.9	3.00	25<br>25<br>25	10<br>10<br>10	38<br>39<br>40	19<br>19<br>20
Paracetamol	152.1	110.0<br>93.1	2.20	25<br>25	10<br>10	22<br>35	53<br>52
Sulfamethoxazole	254.1	156.0<br>108.0<br>92.0	2.80	25<br>25<br>25	10<br>10<br>10	22<br>34<br>38	16<br>12<br>13
Sulfapyridine	249.9	156.0<br>184.0<br>108.0	2.44	25<br>25<br>25	10<br>10<br>10	23<br>25<br>33	10<br>9<br>11
Telmisartan	515.2	497.2<br>276.0	4.12	25<br>25	10<br>10	48<br>61	26<br>17
Tramadol	264.2	58.0<br>42.0<br>246.0	2.92	25<br>25<br>25	10<br>10<br>10	45<br>116<br>16	9<br>16<br>8
Carbamazepine-D10 (IS)	247.1	204.0	3.68	25	10	31	14

Abbreviations:

PhACs – Pharmaceuticals
RT – Retention Time
DP – Declustering Potential
EP – Entrance Potential
CE – Collision Energy
CXP – Collision Cell Exit Potential

Supplementary Table S2. Validation parameters.

PhACs	Spiking level [µg/L]	Average recovery [%]	Coefficient of variation [%]	Extended uncertainty [%]	Calibration curve correlation coefficient
Carbamazepine	0.01	111.5	8.7	3.3	0.998
	0.1	107.0	5.4	2.0	
	0.5	114.3	3.6	4.1	
Diclofenac	0.01	77.1	11.4	6.6	0.995
	0.1	93.7	13.4	1.8	
	0.5	81.3	12.1	5.4	
Fluconazole	0.01	103.9	8.1	1.1	0.999
	0.1	112.6	4.8	3.6	
	0.5	111.1	4.6	3.2	
Gabapentin	0.01	76.1	11.9	6.9	0.995
	0.1	83.1	13.2	4.9	
	0.5	81.7	12.6	5.3	
Lamotrigine	0.01	97.6	13.5	0.7	0.997
	0.1	82.5	14.5	5.0	
	0.5	77.5	12.8	6.5	
Paracetamol	0.01	88.4	14.6	3.3	0.998
	0.1	84.2	11.1	4.6	
	0.5	85.6	3.3	4.2	
Sulfamethoxazole	0.01	111.2	18.3	3.2	0.998
	0.1	111.2	9.3	3.2	
	0.5	103.6	7.8	1.0	
Sulfapyridine	0.01	93.2	13.4	2.0	0.997
	0.1	87.9	15.2	3.5	
	0.5	85.3	10.2	4.2	
Telmisartan	0.01	75.2	10.7	7.2	0.998
	0.1	81.8	13.7	5.2	
	0.5	77.9	16.2	6.4	
Tramadol	0.01	105.9	12.0	1.7	0.999
	0.1	105.2	6.3	1.5	
	0.5	105.3	6.3	1.5	

Three spiking levels, six repetitions for each level, the limit of quantification (LOQ) is the lowest spiking level for which the validation was achieved, six point calibration curve 10, 20, 50, 100, 200 and 500 ng/mL

Supplementary Table S3. Solubility in water, octanol-water partition coefficients (log P) and acid-dissociation constants (pKa) of studied pharmaceuticals.

PhACs	Water solubility [mg/mL]	log P	pKa (strongest acidic)	pKa (strongest basic)
Carbamazepine	0.152	2.77	15.96	-3.8
Diclofenac	0.0045	4.26	4.0	-2.1
Fluconazole	1.39	0.56	12.68	2.3
Gabapentin	4.34	-1.3	4.63	9.91
Lamotrigine	0.488	1.93	14.98	5.89
Paracetamol	4.15	0.91	9.46	-4.4
Sulfamethoxazole	4.459	0.79	6.16	1.97
Sulfapyridine	0.235	1.01	6.24	2.14
Telmisartan	0.0035	6.13	3.62	5.86
Tramadol	0.75	2.45	13.8	9.23
